# Enhanced expression of endogenous retroviruses and of TRIM28 and SETDB1 in children with food allergy

**DOI:** 10.1002/clt2.12124

**Published:** 2022-03-09

**Authors:** Pier‐Angelo Tovo, Giovanna Monti, Valentina Daprà, Paola Montanari, Cristina Calvi, Carla Alliaudi, Allegra Sardo, Ilaria Galliano, Massimiliano Bergallo

**Affiliations:** ^1^ Department of Pediatric Sciences and Public Health University of Turin Turin Italy; ^2^ Pediatric Allergy Unit Regina Margherita Children's Hospital Turin Italy; ^3^ Pediatric Laboratory Department of Pediatric Sciences and Public Health University of Turin Turin Italy

**Keywords:** children, endogenous retroviruses, food allergy, SETDB1, TRIM28

## Abstract

**Background:**

Human endogenous retroviruses (HERVs) represent 8% of our genome. They originate from ancestral infections and although no longer contagious they can regulate transcription of adjacent cellular genes, produce viral RNAs sensed as non‐self by pattern recognition receptors, and encode viral proteins, such as Syncytin (SYN) 1 and 2, that exhibit potent immunomodulatory properties. Based on this, HERVs have been studied and proposed as relevant cofactors in several chronic inflammatory and immune‐mediated diseases. HERV transcription is regulated by host TRIM28 and SET domain bifurcated histone lysine methyltransferase 1 (SETDB1), which in turn exert crucial regulatory functions on the host immune system. No studies explored the expression of HERVs, TRIM28, and SETDB1 in allergic patients.

**Methods:**

We assessed, through a polymerase chain reaction real time Taqman amplification assay, the transcription levels of pol genes of HERV‐H, HERV‐K, HERV‐W, and of env genes of SYN1 and SYN2, as well as of TRIM28 and SETDB1 in whole blood from 32 children with IgE‐mediated food allergy, 19 with food protein‐induced enterocolitis syndrome (FPIES), and in healthy control children.

**Results:**

The expression levels of pol genes of HERV‐H, ‐K, and ‐W were significantly enhanced in patients with IgE‐mediated FA or FPIES as compared to control subjects, while the mRNA concentrations of SYN1 and SYN2 were comparable in each group of children. Both TRIM28 and SETDB1 mRNA levels were significantly higher in allergic patients.

**Conclusions:**

Given the influence of HERVs and of TRIM28 and SETDB1 on innate and adaptive immune responses, their transcriptional activation in children with food allergies suggest that they might play important roles in the development of these diseases.

## 1 INTRODUCTION

Allergic diseases are worldwide among the most common chronic inflammatory disorders, especially in pediatric age. They represent an abnormal reaction to an ordinarily harmless substance referred to as allergen. Food allergy (FA) is an immune‐mediated adverse reaction to specific food(s) with protean clinical manifestations, that is thought to be triggered by a combination of genetic and environmental factors.^1^ It encompasses IgE‐driven and non–IgE‐mediated food‐induced allergic disorders, such as the food protein‐induced enterocolitis syndrome (FPIES).^2^

A key role for the immunological tolerance to foods is thought to be played by dendritic cells (DCs) in the gastrointestinal tract and DCs plus Langerhans cells in the skin, which direct the response of regulatory T cells (Tregs) towards a tolerogenic pattern.^3^, ^4^ In patients with IgE‐mediated FA, the induction of Tregs is altered and it is replaced by a Th2 response leading to the synthesis of food antigen‐specific IgE antibodies.^3^ IL‐33‐driven induction of large amounts of IL‐4 and IgE‐mediated activation of mast cells further contribute to Treg skewing and the amplification of Th2 response.^5^

The immunopathology of FPIES remains elusive.^6^ The syndrome is regarded as a cell‐mediated immune disorder, with food allergens triggering T cell activation that results in abnormal release of proinflammatory cytokines, ultimately leading to intestinal damage.^7^ Recent studies suggest that the innate immune response plays a key role with production of a large array of chemokines and cytokines as well as increased expression of neutrophil and monocyte activation genes.^7^, ^8^

Human endogenous retroviruses (HERVs) originate from ancestral infections that led to their integration into the genome of primates more than 25 millions of years ago.^9^ During evolution, the accumulation of mutations blocked the production of infectious virions and most HERVs became inactive. However, some viral sequences are transcribed and a few encode proteins, such as the Syncytin 1 (SYN1)^10^ and Syncytin 2 (SYN2),^11^ that play crucial roles in placenta morphogenesis and in feto‐maternal tolerance.^12^ HERV elements contribute to the regulation of essential immune functions. They are extensively distributed throughout the human genome and can modulate transcription of close cellular genes.^13^, ^14^ Their RNAs, through retro‐transposition, can generate novel insertions into the genome and, being sensed as non‐self by pattern recognition receptors (PRRs), they can elicit inflammatory and immune reactions.^13^, ^14^, ^15^, ^16^ Furthermore, some viral proteins can trigger autoimmunity,^17^, ^18^ while others, such as the syncytins, exhibit intrinsic immunomodulatory properties.^12^, ^19^, ^20^ Several lines of research have evidenced an association between aberrant HERV expressions and immune‐mediated diseases, supporting the etiopathogenetic role of retroviruses in these pathologies.^18^, ^21^, ^22^, ^23^

Activation of HERVs may be regulated by environmental factors via epigenetic mechanisms, such as DNA methylation and heterochromatin‐silencing by histone modifications. Krüppelassociated box domain zinc finger proteins (KRAB‐ZFPs) are the largest family of transcriptional regulators in the human genome.^24^ Tripartite motif containing 28 (TRIM28), also called KAP1 or TIF1‐β, is a nuclear co‐repressor of KRAB‐ZFPs.^25^ SET domain bifurcated histone lysine methyltransferase 1 (SETDB1), also known as ERG‐associated Protein with a SET domain, is a methyltransferase with high specificity for the lysine 9 residue of histone H3.^26^ Both TRIM28 and SETBD1 represent specific tags for epigenetic transcriptional repression of sequences derived from HERV elements.^27^, ^28^ Additionally, growing data document their involvement in many aspects of cell homeostasis and in epigenetic control of both innate and adaptive immune responses.^29^, ^30^ Notably, accumulating evidence highlights the importance of epigenetic mechanisms in the pathogenesis of allergic diseases,^31^, ^32^ including FA.^33^, ^34^

The understanding of underlying pathophysiology and the identification of molecules involved in the development of FA may contribute to its better prevention and to innovative treatment modalities.^35^ Despite the potential impact of HERVs and of TRIM28 and SETDB1 in triggering and/or maintaining allergic reactions, to the best of our knowledge no studies explored their expressions in allergic patients. The aims of the current study were to assess the transcription levels of pol genes of HERV‐H, ‐K, and ‐W, the three retroviral families most widely studied,^9^, ^21^ of env genes of SYN1 and SYN2 as well as of TRIM28 and SETDB1 in whole blood from children affected by IgE‐mediated FA or FPIES and in control healthy children.

## 2 MATERIALS AND METHODS

### 2.1 Study populations

Three groups of children were investigated: subjects with IgE‐mediated FA (Group A), subjects with FPIES (Group B), and control healthy subjects (Group C).

Group A and Group B children were enrolled at Pediatric Allergy Unit of the Regina Margherita Children's Hospital, Turin, Italy. They were tested after suspension from at least 1–3 months of the triggering food(s) during a routine laboratory control. At time of testing, all subjects were in good general condition, had no symptoms or signs of allergy, and inflammatory markers (WBC count, C reactive protein, erythrocyte sedimentation rate) were all within the normal range.

The Group C included asymptomatic children who were tested at the Regina Margherita Children's Hospital for routine laboratory examinations and whose results were all within the normal reference range. Subjects with any confirmed or suspected disease, such as allergy, infections, cancer, autoimmune disorders, inflammatory diseases, neurological disturbances, or abnormal laboratory results were excluded from the study.

### 2.2 Diagnostic criteria

The diagnosis of IgE‐mediated FA was based on the personal history of acute allergic symptoms (cutaneous, ocular, upper/lower respiratory, oral/lower gastrointestinal, cardiovascular, anaphylaxis) appearing within 1 h after ingestion of a specific food and confirmed by oral food challenge (except for severe anaphylaxis), a positive skin prick test and/or a serum food‐specific IgE level of ≥ 0.10 kU/l. When available, sIgE antibodies against food molecules were also investigated (component‐resolved diagnostic tests [CRD]).^36^, ^37^

FPIES was diagnosed according to the international consensus guidelines for its diagnosis and management.^38^

### 2.3 Total RNA extraction

Total RNA was extracted from whole blood using the automated extractor Maxwell (Promega, Madison, WI, USA) following the RNA Blood Kit protocol without modification. This kit provides treatment with DNase during the RNA extraction process. RNA concentration and purity were assessed by traditional UV spectroscopy with absorbance at 260 and 280 nm. The nucleic acid concentration was calculated using the Beer‐Lambert law, which predicts a linear change in absorbance with concentration. The RNA concentration range was within manufacturer specifications for the NanoDrop (Thermofisher Scientific). UV absorbance measurements were acquired using 1 µl of RNA sample in an ND‐1000 spectrophotometer under the RNA‐40 settings at room temperature (RT). Using this equation, an A260 reading of 1.0 is equivalent to ∼40 µg/ml single‐stranded RNA. The A260/A280 ratio was used to define RNA purity. An A260/A280 ratio of 1.8/2.1 is indicative of highly purified RNA. RNA extracts were directly amplified without reverse transcription to control the genomic DNA contamination. The RNAs were stored at −80° until use.

### 2.4 Reverse transcription

Four hundred nanograms of total RNA were reverse‐transcribed with 2 μl of buffer 10X, 4.8 μl of MgCl_2_ 25 mM, 2 μl ImpromII (Promega), 1 μl of RNase inhibitor 20U/l, 0.4 μl random hexamers 250 μM (Promega), 2 μl mix dNTPs 100 mM (Promega), and dd‐water in a final volume of 20 μl. The reaction mix was carried out in a GeneAmp polymerase chain reaction (PCR) system 9700 Thermal Cycle (Applied Biosystems) under the following conditions: 5 min at 25°C, 60 min at 42°C and 15 min at 70°C for the inactivation of enzyme; the cDNAs were stored at −80° until use.

### 2.5 Transcription levels of pol genes of HERV‐H, ‐K, and ‐W, of env genes of Syn1, Syn2, and of TRIM28 and SETB1 by real‐time PCR assay

Glyceraldehide 3‐phosphate dehydrogenase (GAPDH) was chosen as reference gene in all determinations being one of the most stable among reference genes and already used in our previous studies.^22^, ^23^, ^39^, ^40^, ^41^ Relative quantification (RQ) of mRNA concentrations of HERV‐H‐pol, HERV‐K‐pol, HERV‐W‐pol, SYN1‐env, SYN2‐env, TRIM28, and SETB1 was achieved by using the ABI PRISM 7500 real time system (Thermofisher Scientific).

Forty ng of cDNA were amplified in a 20 μl total volume reaction using HERV‐H, ‐K ‐W mRNA expression kit PP‐054, ‐055, and ‐056, respectively (BioMole). The PP‐BioMole‐055 was derived from Schanab et al.^42^ The SYN1‐env and SYN2‐env mRNA expressions were also quantified by real‐time PCR. Forty ng cDNA were amplified in a 20 μl of total volume reaction containing 2.5 U goTaQ MaterMix (Promega), 1.25 mmol/l MgCl_2_, 500 nmol of specific primers and 200 nmol of specific probes. The SYN1 primers were: (Sinc1F 5′‐ACTTTGTCTCTTCCAGAATCG‐3′) (Sinc1R 5′‐GCGGTAGATCTTAGTCTTGG‐3′), and the probe was: (Sinc1P 6FAM‐TGCATCTTGGGCTCCAT‐TAMRA).^43^ The Syn 2 primers were: (Sinc2F‐GCCTGCAAATAGTCTTCTTT‐3′) (Sinc2R‐ ATAGGGGCTATTCCCATTAG‐3′),^44^ and the probe was: (Sinc2P‐6FAM‐ TGATATCCGCCAGAAACCTCCC‐TAMRA) (this study). The probes were designed by Primer ExpressTM software version 3.0 (Applied Biosystems).

For TRIM28 and SETDB1 detection, 40 ng of cDNA were amplified using mRNA expression kit PP‐044 and PP‐045, respectively (BioMole), in a 20 μl total volume reaction.

The amplifications were run in a 96‐well plate at 95°C for 10 min, followed by 45 cycles at 95°C for 15 s and at 60°C for 1 min. Each sample was run in triplicate. RQ of target gene transcripts was performed with the ΔΔCt method. Hence, fold change was calculated and results were expressed in corresponding arbitrary units, called RQ. Since we measured Ct for every target in all samples, we argued that our methods were suitable for HERV detection and quantification.

### 2.6 Statistical analysis

One‐way analysis of variance (ANOVA) test was used to compare the transcriptional levels of pol genes of HERV‐H, ‐K, and ‐W, of env genes of SYN1, SYN2, and of TRIM28 and SETDB1 between the three groups of children. Mann‐Whitney test was used to compare the transcripts of pol genes of every HERV family as well as of SYN1, SYN2, TRIM28, and SETDB1 between each group of children. Mann‐Whitney test was used to compare the transcription levels of HERVs between males and females. Spearman correlation test was used to evaluate the correlations between age and the transcription levels of each HERV sequence and of TRIM28 and SETDB1. Statistical analyses were done using the Prism software (GraphPad Software). In all analyses, *p* < 0.05 was taken to be statistically significant.

## 3 RESULTS

### 3.1 Study populations

The characteristics of children affected by IgE‐mediated FA or FPIES are reported in Table 1. In particular, Group A included 32 subjects with IgE‐mediated FA and Group B 19 children with FPIES. Group C encompassed 3 subgroups of healthy control subjects: Group C1 included 78 children (38 males, median age 4.1 years, interquartile range (IQR) 3.2, 5.5 years) who had been investigated as controls in our previous studies on pol gene expressions of HERV‐H, ‐K, and ‐W,^31^, ^32^, ^55^ Group C2 included 31 children who were tested for SYN1 and SYN2 expressions (22 males, median age 2.0 years, IQR 1.1, 4.2 years), and Group C3 included 34 children evaluated for TRIM28 and SETDB1 expressions (24 males, median age 6.1 years, IQR 4.1, 7.9 years).

**TABLE 1:** Characteristics of children with food protein‐induced enterocolitis syndrome (FPIES) and with IgE‐mediated food allergy (IgE‐FA)

	FPIES (*n* = 19)	IgE‐FA (*n* = 32)
Family history of atopy: *n* (%)	11 (58)	32 (100)
Males: *n* (%)	7 (36.8)	19 (59.4)
Age at testing (years): median (IQR)	2.6 (2.0–5.2)	10.0 (7.0–12.2)
Age at allergy onset (years): median (IQR)	0.67 (0.33–1.23)	0.75 (0.36–1.27)
History of atopic comorbidities: *n* (%):		
Atopic dermatitis	5 (26.3)	23 (72)
Anaphylaxis episodes	0 (0)	21 (65.6)
Pollen allergy	0 (0)	14 (43.8)
Trigger food(s): *n* (%):		
Egg	9 (47.4)	16 (50)
Milk	5 (26.3)	12 (37.5)
Fish	5 (26.3)	6 (18.7)
Wheat	1 (5.3)	6 (18.7)
Other grains	0	2 (6.2)
Nuts	0	15 (47)
Peanut	0	5 (15.6)
Fruits	1 (5.3)	4 (12.5)
Legumes	0 (0)	4 (12.5)
Poultry	3 (15.8)	0 (0)
Others	4 (21)	2 (6.2)

Abbreviations: IQR = interquartile range 25%–75%, n = number.

### 3.2 Expression levels of housekeeping gene

Transcription levels of housekeeping gene GAPDH were similar between each group of children. Group A (median, IQR): 21.52, 21.14–21.99; Group B: 21.36, 21.01–21.81; Group C1: 21.00, 20.81–21.58; Group C2: 21.86, 21.48–22.27; and Group C3: 21.04, 20.80–21.52.

### 3.3 Influence of age and gender on transcription levels of HERVs, TRIM28, and SETDB1

The median age was significantly different in the three groups of children. In particular, patients with IgE‐mediated FA were older than children of the control groups (*p* < 0.0001 for Group C1; *p* < 0.0001 for Group C2; *p* = 0.0011 for Group C3) and of patients with FPIES (*p* = 0.0007). These were younger than children of C1 and C3 control groups (*p* = 0.0349 and *p* = 0.0011, respectively) while their age was comparable to C2 children (*p* = 0.1398).

**FIGURE 1:**
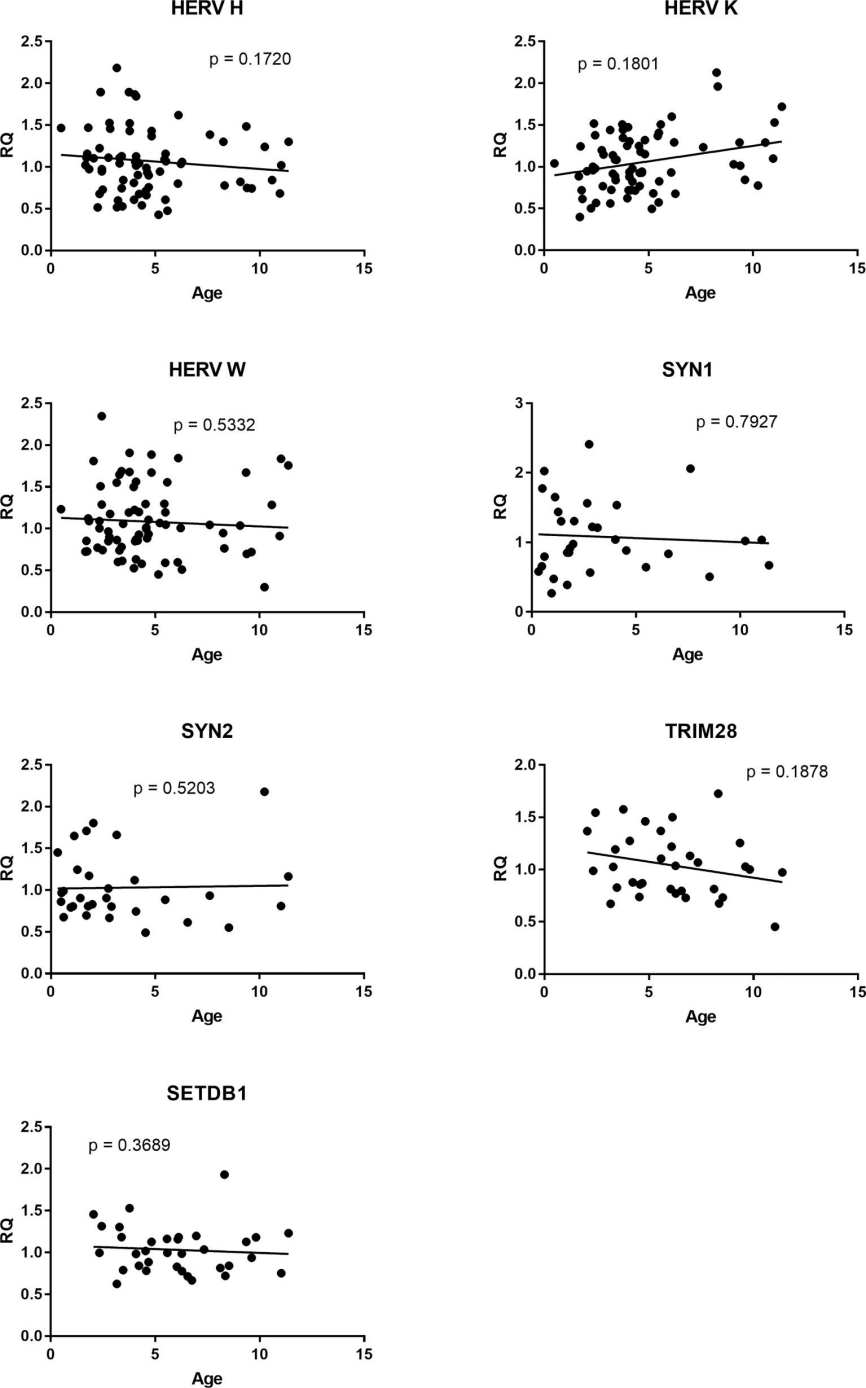
Correlations between age and transcription levels of pol genes of HERV‐H, ‐K, ‐W, Syncytin (SYN)‐1‐env, SYN‐2‐env, SET domain bifurcated histone lysine methyltransferase 1 (SETDB1), and TRIM28 in whole blood from control children. Circles show the mean of three individual measurements. Line: Linear regression line. Statistical analysis: Spearman correlation test. HERV, human endogenous retrovirus; RQ, relative quantification

The transcriptional levels of each target gene were however not related to the age. As illustrated in Figure 1, no significant correlations were observed between age and single gene expressions in the control groups. The lack of correlation between age and transcriptional levels of HERVs, TRIM28 and SETDB1 emerged also in Group A plus Group B patients (age vs. HERV‐H‐pol: *p* = 0.2683; age vs. HERV‐K‐pol: *p* = 0.7516; age vs. HERV‐W‐pol: *p* = 0.3215; age vs. SYN1 *p* = 0.5916; age vs. SYN2 *p* = 0.9282; age vs. TRIM28 *p* = 0.2211; age vs. SETDB1 *p* = 0.9690).

**FIGURE 2:**
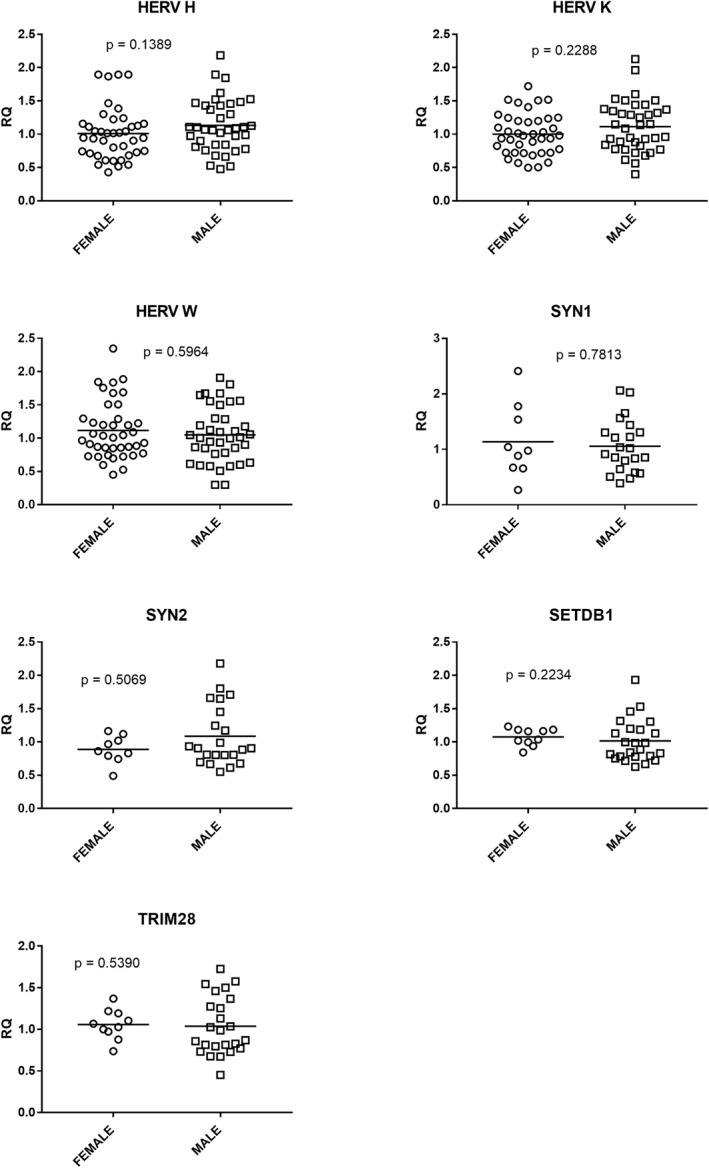
Transcription levels of pol genes of HERV‐H, ‐K, ‐W, of Syncytin (SYN)1‐env, SYN2‐env, TRIM28, and SET domain bifurcated histone lysine methyltransferase 1 (SETDB1) in females and males of control children. Median, IQR 25%–75%: HERV‐H pol: females 0.98, 0.72–1.16, males 1.08, 0.84–1.43; HERV‐K pol: females 0.98, 0.72–1.21, males 1.11, 0.83–1.36; HERV‐W pol: females 0.88, 0.72–1.19, males 1.11, 0.91–1.63; SYN1‐env: females 0.98, 0.67–1.54, males 0.97, 0.68–1.31; SYN2‐env: females 0.86, 0.79–1.02, males 0.91, 0.80–1.40; TRIM28: females 1.05, 0.98–1.17, males 0.93, 0.79–1.30; SETDB1: females 1.10, 1.00–1.18, males 0.94, 0.78–1.19. Circles and squares show the median of three individual measurements, horizontal lines the median values. Statistical analysis: Mann–Whitney test was used to compare the transcriptional levels of the target genes between females and males. HERV, human endogenous retrovirus; IQR, interquartile range; RQ, relative quantification

Figure 2 shows that the transcription levels of each target gene were not related to the gender in control subjects. Similar findings emerged in Group A + Group B patients: Median, IQR 25%–75%: HERV‐H‐pol: females 1.69, 130–2.05, males 1.60, 1,34–2.21, *p* = 0.9182; HERV‐K‐pol: females 2.02, 1.39–2.27, males 1.46, 1.07–1.96, *p* = 0.1795; HERV‐W‐pol: females 1.71, 1.35–1.94, males 1.57, 1.38–1.99, *p* = 0.7259; SYN1‐env: females 0.84, 0.65–1.24, males 0.75, 0.50–0.94, *p* = 02264; SYN2‐env: females 0.98, 0.69–1.31, males 0.72, 0.44–1.10, *p* = 0.1857; TRIM28: females 1,43, 1.03–1.81, males 1.23, 089–1.42, *p* = 0.1219; SETDB1 females 1.94, 1.40–2.34, males 1.67, 1.42–2.06, *p* = 0.2668.

### 3.4 Transcription levels of HERV‐H‐pol, HERV‐K‐pol, HERV‐W‐pol, SYN1‐env, and SYN2‐env in children with FA and control children

**FIGURE 3:**
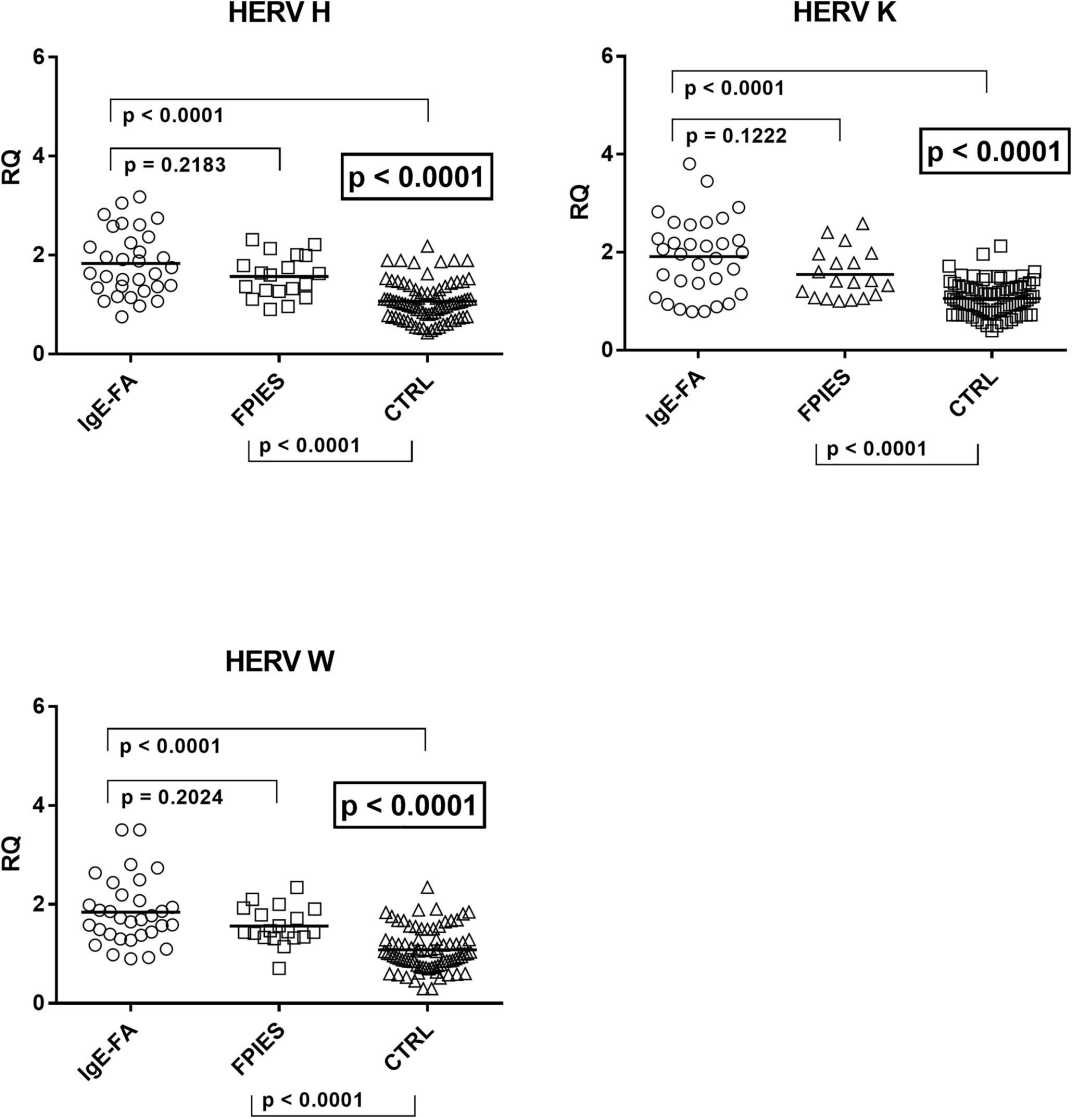
Transcription levels of pol genes of HERV‐H, ‐K, ‐W in whole blood from children with IgE‐mediated FA, with food protein‐induced enterocolitis syndrome (FPIES), and from control children (CTRL). Median, IQR 25%–75%: HERV‐H‐pol: Group A 1.69, 1.36–2.28; Group B 1.60, 1.28–1.89; Group C1 1.03, 0.76–1.29; HERV‐K‐pol: Group A 1.98, 1.31–2.35; Group B 1.41, 1.10–1.88; Group C1 0.99, 0.78–1.29; HERV‐W‐pol: Group A 1.71, 1.39–2.10; Group B 1.45, 1.34–1.85; Group C1 1.01, 0.77–1.29. Circles, squares, and triangles show the median of three individual measurements, horizontal lines the median values. Statistical analysis: One‐Way ANOVA was used to compare the transcriptional levels of HERVs between the three groups of children. Mann‐Whitney test was used to compare values of each group of children with each other. ANOVA, analysis of variance; HERV, human endogenous retrovirus; IQR, interquartile range; RQ, relative quantification

The ANOVA analysis showed that there was a statistically significant difference in the transcription levels of pol genes of HERV‐H, ‐K, and ‐W between the three groups of subjects (Figure 3). As detailed in the figure children with IgE‐mediated FA or with FPIES had significantly higher values than the control group for HERV‐H pol while no difference emerged between Group A and Group B patients. Overlapping results were observed in the transcription levels of pol genes of HERV‐K and HERV‐W. In particular, HERV‐K‐pol values were significantly higher in children with both IgE‐mediated FA and with FPIES as compared to the control group, while no difference was found between Group A and Group B patients (Figure 3). Similar results were found for HERV‐W‐pol, whose values were significantly higher in children with IgE‐mediated FA or with FPIES than in the control group, while no difference emerged between Group A and Group B patients (Figure 3).

**FIGURE 4:**
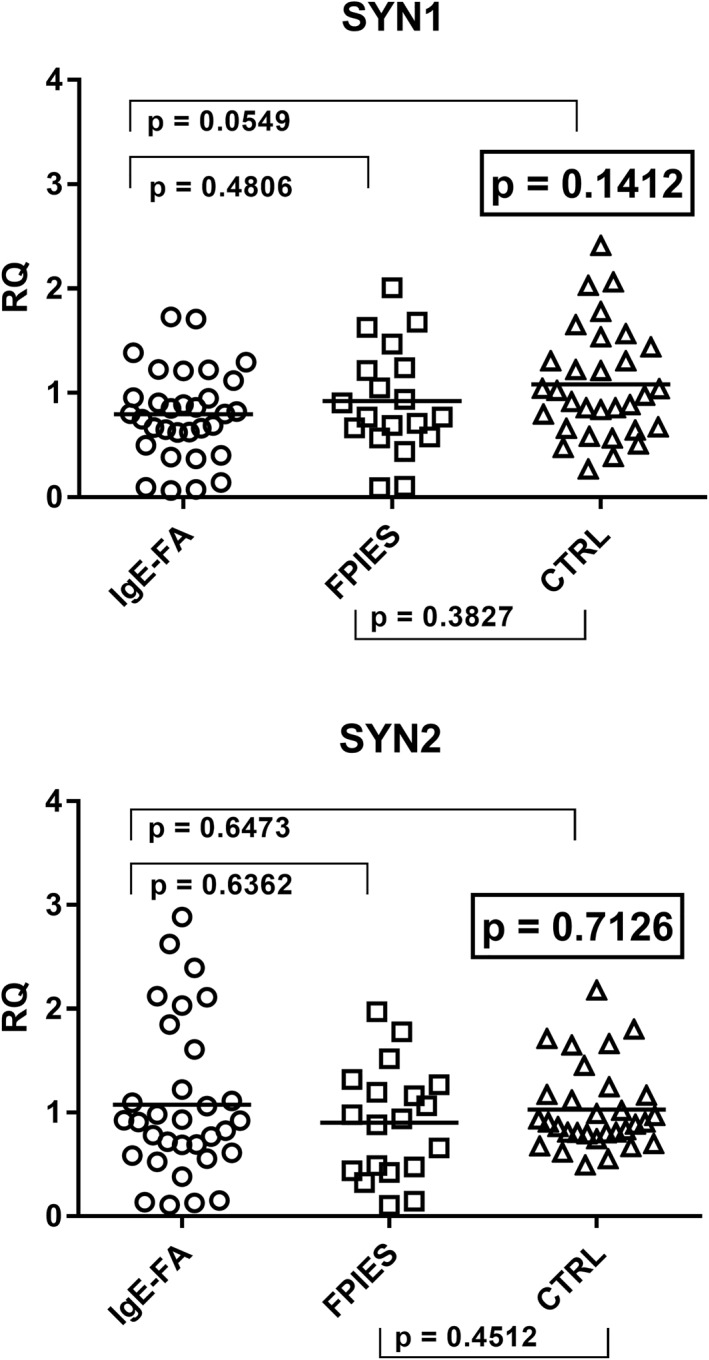
Transcription levels of Syncytin (SYN)1‐env and SYN2‐env in whole blood from children with IgE‐mediated food allergy (IgE‐FA), with food protein‐induced enterocolitis syndrome (FPIES), and from control children (CTRL). Median, IQR 25%–75%: Syn1‐env: Group A 0.80, 0.59–0.99; Group B 0.77, 0.62–1.23; Group C2 0.98, 0.66–1.37; Syn2‐env: Group A 0. 91, 0.60–1.32; Group B 0.94, 0.46–1.23; Group C2 0.90, 0.80–1.17. Circles, squares, and triangles show the median of three individual measurements, horizontal lines the median values. Statistical analysis: One‐Way ANOVA was used to compare the transcriptional levels of SYN‐1 and SYN‐2 between the three groups of children. Mann‐Whitney test was used to compare values of each group of children with each other. ANOVA, analysis of variance; IQR, interquartile range; RQ, relative quantification

In contrast, as illustrated in Figure 4, no significant differences were observed in the mRNA concentrations of SYN1‐env and SYN2‐env between the three groups of children. .

### 3.5 Transcription levels of TRIM28 and SETDB1 in children with FA and control children

**FIGURE 5:**
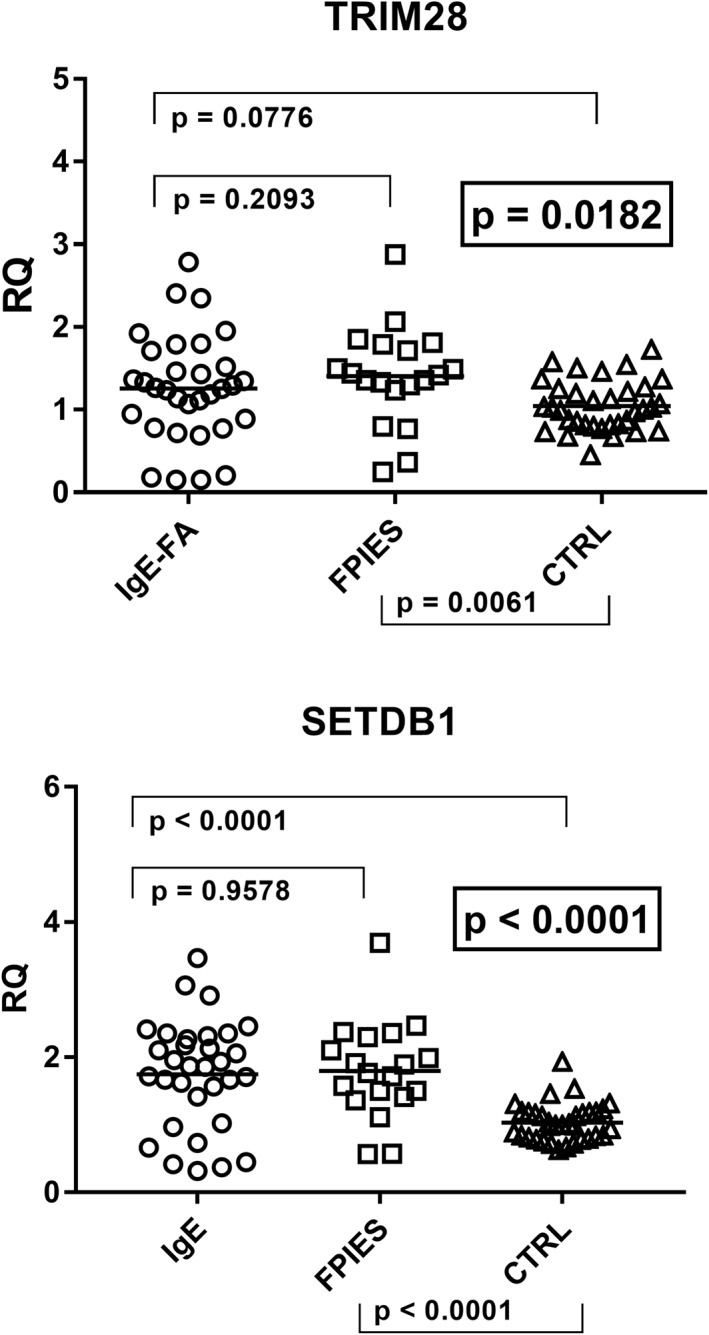
Transcription levels of TRIM28 and SET domain bifurcated histone lysine methyltransferase 1 (SETDB1) in whole blood from children with IgE‐mediated food allergy (IgE‐FA), with food protein‐induced enterocolitis syndrome (FPIES), and from control children (CTRL). Median, IQR 25%–75%: TRIM28: Group A 1.26, 0.86–1.57; Group B 1.42, 1.27–1.75, Group C3 1.01, 0.81–1.24; SETDB1: Group A 1.86, 1.32–2.28; Group B 1.77, 1.46–2.20; Group C3 1.00, 0.82–1.18. Circles, squares, and triangles show the median of three individual measurements, horizontal lines the median values. Statistical analysis: One‐Way ANOVA was used to compare the transcriptional levels of TRIM28 and SETDB1 between the three groups of children. Mann‐Whitney test was used to compare values of each group of children with each other. ANOVA, analysis of variance; IQR, interquartile range; RQ, relative quantification

The ANOVA analysis showed that there were statistically significant differences in the transcription levels of TRIM28 and SETDB1 between the three groups of subjects (Figure 5). As reported in the figure, the transcription levels of TRIM28 were significantly higher in children with FPIES than in the control group, with borderline *p* value for children with IgE‐mediated FA and control children, while no difference was found between Group A and Group B patients.

SETDB1 mRNA concentrations were significantly enhanced in children with IgE‐mediated FA or with FPIES as compared to the control group, while no difference emerged between Group A and Group B patients (Figure 5).

## 4 DISCUSSION

Present results document, for the first time, significantly higher transcriptional levels of pol genes of HERV‐H, HERV‐K, and HERV‐W and of TRIM28 and SETDB1 in children affected by IgE‐mediated FA or FPIES as compared to healthy children.

Reactions of FPIES typically manifest during the first years of life. This accounts for the younger age of these patients as compared to control subjects or patients with IgE‐mediated FA. However, in control children the age did not influence the expression of any target gene and similar findings were observed in allergic patients. Therefore, the impact of the age on the trans‐activation of the genes here analyzed was irrelevant. No significant differences emerged also between males and females, in line with lack of association between sex and FA.^45^

In contrast to HERV‐pol sequences, both SYN1‐env and SYN2‐env mRNA levels were comparable in the three groups of subjects. There is a general agreement that syncytins exert important regulatory functions on a large array of immune responses and in the induction of immune‐tolerance.^12^, ^14^, ^19^, ^20^ However, the normal expression profiles of SYN1 and SYN2 in children with IgE‐mediated FA or FPIES suggest that they do not play an important role in the pathogenesis of these diseases.

The underlying biochemical mechanisms responsible for the enhanced activation of pol genes of every HERV family in children with FA and their potential clinical significance remain to be elucidated. TRIM28 e SETDB1 are potent corepressors of retroviruses. Their higher expressions result in enhanced DNA methylation and heterochromatin formation ultimately leading to HERV silencing.^27^, ^28^ However, TRIM28 and SETDB1 mRNA concentrations were unexpectedly not reduced, but enhanced in both groups of allergic patients. Therefore, the enhanced HERV‐pol activation cannot be ascribed to impaired transcription of TRIM28 or SETDB1 repressors. It is worth noting that no reduced TRIM28/SETDB1 expression was found also in other immune‐mediated diseases characterized by increased levels of HERV‐pol mRNAs, such as in new‐onset type 1 diabetes^22^ and celiac disease.^23^

Allergy is thought to originate from a dysfunction of the immune system. As mentioned, growing evidence indicates that endogenous retroviruses can condition and shape the host immune response. There is wide consensus on the crucial role played by forkhead box p3+ (Foxp3) Tregs to ensure immunological tolerance, while their dysfunctions may lead to allergic reactions,^46^, ^47^ including FA.^48^ In mice, retroviral superantigens have been shown to induce expansion and activation of Foxp3+ Tregs.^49^ This notwithstanding, whether HERVs are really responsible for immune‐mediated damages or their activation represents a secondary epiphenomenon remains an unsolved dilemma. Inflammatory‐driven activation of NF‐kB and production of pro‐inflammatory cytokines can elicit HERV transcription.^50^ On the other hand, HERVs can in turn induce flogosis,^9^, ^16^, ^21^ giving rise to a vicious circle to trigger local and systemic inflammatory responses. In this context, it must be underlined that in our patients HERV upregulation was detected after suspension of the culprit foods from several weeks and they were asymptomatic with normal inflammatory markers at time of sampling. This suggests that the enhanced HERV‐pol transcriptions in subjects with IgE‐mediated FA or FPIES were not sustained by ongoing allergic inflammation, although a long‐lasting local reaction cannot be excluded.

Our results on HERV‐pol expressions have some technical limits which did not allow to better characterize the potential involvement of HERV copies with transcriptional activity, in particular the contribution of specific HERV loci. Furthermore, we did not assess their protein‐coding capacity.

Allergic diseases, including FA, are thought to be multifactorial disorders triggered by complex interactions between genetic and environmental factors. The69 impact of the latter may occur via epigenetic alterations, such as DNA methylation and histone modifications.^31^, ^32^ Epigenome‐wide association studies linked specific leukocyte DNA methylation patterns with FA.^33^, ^34^ In peanut‐sensitized mice DNA methylation changes at specific T cell loci gave evidence to the antiallergic protection of epicutaneous immunotherapy.^51^ In patients with peanut allergy the treatment with oral immunotherapy resulted in increased antigen‐driven Treg function and hypomethylation of Foxp3. This persisted in subjects who remained clinically non‐reactive, while those who re‐sensitized returned to higher Foxp3 methylation state.^52^ TRIM28 and SETDB1 regulate the expression of thousands of target genes.^53^, ^54^ TRIM28 is essential for T cell development, activation, and through a complex with KRAB‐ZFP and Foxp3 modulates Treg suppressor activity.^55^, ^56^, ^57^ Furthermore, it represses activation of inflammatory genes, while its deficiency increases expansion of DCs and enhanced T cell priming toward inflammatory effector T cells.^55^ SETDB1 has multifaced biological properties. It controls Th1 gene network and ensures Th2 cell stability via its action on a set of retroviral elements close to genes involved in immune response.^58^ In addition, SETDB1 is essential for intestinal epithelial homeostasis and prevention of local inflammation.^59^, ^60^ Taken together these findings suggest that the aberrant expressions of TRIM28 and SETDB1 in children with IgE‐mediated FA or FPIES might mirror their actions in directing the epigenetic differentiation, expansion, and function of DC and T cells towards the peculiar reactivity to food allergens in genetically predisposed subjects.

The number of patients with allergic disturbances has dramatically increased over the past decades.^3^ The reason of this higher prevalence of allergies remains ill‐defined. A major role is attributed to the influence of external factors. Pollutants, chemicals, nutrients or changes in life style (the so‐called external exposome) have been identified as risk factors for allergy, with their action being mediated, at least in part, by epigenetic mechanisms.^61^ TRIM28 and SETDB1 play pivotal roles in inducing epigenetic changes. Furthermore, the same environmental factors, such as pollution, for example exposure to pesticides^62^ or cigarette smoking,^39^ or nutritional changes linked to the life style,^63^ display a significant impact on retrovirus expression. Therefore, environmental factors responsible for the increasing number of subjects affected by allergy could exert their actions via TRIM28/SETDB1‐ and/or HERV‐driven variations in targeted biologic processes.

Our findings raise further intriguing questions. Are overexpressions of HERVs and/or of TRIM28 and SETDB1 biomarkers of FA? Do they precede the challenge with the culprit food? Do they persist over time, for instance in children with FPIES when their food‐induced reaction disappears? One wonders whether also subjects affected by other allergies or asthma show enhanced expressions of these variables. What happens in case of antiallergic protection given by specific immunotherapy? In conclusion, given the strict interactions between HERVs, TRIM28, and SETDB1 and the immune system, our results may open new avenues of research focusing on retroviruses and epigenetic regulatory pathways not only to better understand the etiopathogenesis of allergy, but also as potential innovative diagnostic and therapeutic targets.

## DECLARATIONS

The study was carried out in accordance with the principles of Helsinki Declaration (World Medical Association General Assembly, Seoul, Korea, October 2008). The study protocol was approved by the ethics committee of the Azienda Ospedaliera‐Universitaria Città della Salute e della Scienza, Turin (code n°59884). Blood samples were collected from leftovers of laboratory samples after parent's informed consent. Data were gathered anonymously.

## CONFLICT OF INTEREST

The authors declare no conflict of interest.

## AUTHOR CONTRIBUTIONS

Pier‐Angelo Tovo, substantial contributions to the conception of the work; drafting the work, revising it critically for important intellectual contents; G.M. enrollment and follow‐up of patients, drafting the work, revising it critically for important intellectual contents; Valentina Daprà, Cristina Calvi, Carla Alliaudi, Paola Montanari, Ilaria Galliano, performed laboratory tests, substantial contributions to analysis and interpretation of data; Allegra Sardo contributions to enrollment and follow‐up of patients; Massimiliano Bergallo, substantial contributions to the conception and design of the work, the acquisition, analysis, and interpretation of data, and drafting of the manuscript. All authors read and approved the final manuscript.
